# Effects of Omega-3 Supplementation on Inflammatory Biomarkers and Ambulatory and Central Blood Pressure in Patients Treated for Hypertension and Dyslipidemia: A Randomized, Double-Blind, Placebo-Controlled Trial

**DOI:** 10.3390/nu18213717

**Published:** 2026-11-09

**Authors:** Carol Kotliar, Rocío Martínez Vivot, Cristian Beltramo, Ezequiel Huguet, Alfredo Lozada, Laura Montecchiesi, Leopoldo Marcon, Sebastián Eduardo Obregón

**Affiliations:** 1 Santa María de la Salud Medical Center, Av. del Libertador 16131, San Isidro 1643, Buenos Aires, Argentina; cristianbeltramo@hotmail.com (C.B.); ezehuguet@yahoo.com.ar (E.H.); aflozada@yahoo.com.ar (A.L.); lalimontecchiesi@gmail.com (L.M.); leomarcon@hotmail.com (L.M.); sobregon@cas.austral.edu.ar (S.E.O.); 2 Institute for Biomedical Research, School of Medical Sciences, Pontifical Catholic University of Argentina, National Scientific and Technical Research Council, Alicia Moreau de Justo 1600, Capital Federal, Buenos Aires 1107, Argentina; rmartinezvivot@gmail.com; 3 Center of Hypertension, Institute of Cardiology, Hospital Universitario Austral, Av.Perón 1500, Pilar 1629, Buenos Aires, Argentina; 4 Cholesterol Clinic, Hospital Universitario Austral, Av.Perón 1500, Pilar 1629, Buenos Aires, Argentina

**Keywords:** omega-3 fatty acids, EPA, DHA, hypertension, dyslipidemia, inflammation, neutrophil-to-lymphocyte ratio, ambulatory blood pressure, central blood pressure, pulse wave velocity

## Abstract

Background: Omega-3 fatty acids exert anti-inflammatory and hemodynamic effects; however, their concurrent effects in patients receiving treatment for hypertension and dyslipidemia remain incompletely characterized. Objective: To evaluate the effects of 12 weeks of supplementation with pharmaceutical-grade omega-3 fatty acids on inflammatory biomarkers, central and brachial blood pressure including office and 24 h ambulatory measurements, and vascular function in this population. Methods: In this exploratory, randomized, double-blind, placebo-controlled pilot trial, 100 adults received omega-3 ethyl esters at a capsule dose of 2 g/day or a matching placebo for 12 weeks, in addition to stable usual pharmacological treatment. Treatment effects were estimated using linear mixed-effects models. *p*-values were nominal, and 95% confidence intervals were not adjusted for multiplicity. Results: Compared with placebo, omega-3 supplementation was associated with greater reductions in the neutrophil-to-lymphocyte ratio (NLR; −0.43; 95% CI, −0.73 to −0.13; *p* = 0.005), ferritin (−47.32 ng/mL; 95% CI, −77.55 to −17.08; *p* = 0.003), daytime systolic blood pressure (−6.52 mmHg; 95% CI, −10.66 to −2.39; *p* = 0.002), nighttime diastolic blood pressure (−5.55 mmHg; 95% CI, −9.87 to −1.22; *p* = 0.013), central systolic blood pressure (−6.71 mmHg; 95% CI, −12.22 to −1.20; *p* = 0.019), and estimated pulse wave velocity (−0.77 m/s; 95% CI, −1.19 to −0.36; *p* < 0.001). Conclusions: The trial identified exploratory signals involving NLR, ferritin, selected ambulatory blood-pressure measures, central systolic blood pressure, and algorithm-derived ePWV. Multiplicity, limited sample size, and incomplete outcome availability preclude confirmatory interpretation. Independent replication in larger, prospectively registered trials with clearly specified endpoints and reproducible sample-size calculations is required.

## 1. Introduction

Hypertension and dyslipidemia frequently coexist and increase cardiovascular risk through overlapping inflammatory, hemodynamic, vascular, and metabolic mechanisms. Their coexistence is associated with endothelial dysfunction, altered vascular tone, oxidative stress, and pro-inflammatory signaling, supporting an integrated approach to cardiovascular risk rather than treatment of each risk factor in isolation [1,2].

Long-chain omega-3 polyunsaturated fatty acids have attracted considerable interest as potential adjunctive therapies for addressing persistent inflammatory and hemodynamic abnormalities in patients receiving standard cardiometabolic treatment [3,4,5]. Eicosapentaenoic acid (EPA) and docosahexaenoic acid (DHA) exert multiple biological effects that extend beyond lipid metabolism, including modulation of inflammatory signaling pathways, regulation of leukocyte function, attenuation of pro-inflammatory cytokine production, and generation of specialized pro-resolving mediators such as resolvins, protectins, and maresins [3,4].

Clinical evidence also supports modest blood-pressure-lowering effects of EPA + DHA. A dose–response meta-analysis of 71 randomized controlled trials involving 4973 participants found an overall inverse association between omega-3 intake and both systolic and diastolic blood pressure, with effects varying according to dose and participant characteristics [5].

The effects of omega-3 fatty acids on systemic inflammation are less uniform across studies and biomarkers. An umbrella meta-analysis of 32 meta-analyses reported reductions in circulating C-reactive protein, tumor necrosis factor-α, and interleukin-6 following n-3 PUFA supplementation, but also substantial heterogeneity across studies, populations, and inflammatory outcomes [6]. These observations are consistent with the biological complexity of EPA- and DHA-mediated immunomodulation and suggest that the inflammatory response to supplementation may not be adequately characterized by a single circulating biomarker [3,4].

Although the effects of omega-3 supplementation on conventional lipid and blood-pressure outcomes have been extensively studied, inflammatory, hemodynamic, and vascular responses have often been evaluated separately. Less is known about their concurrent effects in pharmacologically treated patients in whom hypertension and dyslipidemia coexist, particularly when blood pressure is characterized beyond conventional office measurements. Ambulatory blood pressure monitoring provides information complementary to office blood pressure, with 24 h and nighttime blood pressure showing particularly strong associations with cardiovascular outcomes [7]. Central systolic blood pressure may provide additional information on cardiovascular load and has been associated with cardiovascular events independently of peripheral systolic blood pressure [8]. Evaluating ambulatory and central hemodynamics together with vascular indices and individual inflammatory biomarkers may therefore provide a broader characterization of the physiological response to omega-3 supplementation in this clinical setting.

The aim of this study was to evaluate the effects of 12 weeks of EPA + DHA supplementation, compared with placebo, on inflammatory biomarkers, blood pressure-hemodynamic parameters, and vascular function in treated patients with hypertension and dyslipidemia. We hypothesized that omega-3 supplementation would improve blood pressure control and vascular function and reduce inflammatory biomarker levels compared with placebo.

## 2. Materials and Methods

### 2.1. Study Design and Participants

This was an exploratory, randomized, double-blind, placebo-controlled pilot trial evaluating the short-term effects of 12 weeks of pharmaceutical-grade omega-3 ethyl ester supplementation, administered as an adjunct to stable usual pharmacological treatment, in adults with hypertension and dyslipidemia.

Participants were recruited from the Cardiometabolic Clinic, Santa María de la Salud Academic and Medical Center, in Buenos Aires, Argentina.

Eligible participants were men and women aged 21–70 years with diagnosed arterial hypertension and dyslipidemia who were receiving pharmacological treatment stable for at least 3 months as confirmed by their current treating physician and clinical record. Exclusion criteria included known allergy to fish, seafood, soy, or coconut oil; current omega-3 supplementation; hepatic insufficiency; anticoagulant therapy; and any foreseeable circumstance that could prevent completion of study follow-up.

### 2.2. Sample Size

A target sample size of 100 participants (50 participants per treatment group) was prospectively established in the study protocol. The original sample-size rationale was based on an anticipated 10% post-intervention versus baseline reduction in blood-pressure parameters, including mean arterial pressure, brachial systolic and diastolic blood pressure, and/or central systolic blood pressure. The statistical section of the available protocol version reports a two-sided alpha of 0.05, 80% power, an anticipated standardized effect size of approximately 0.5–0.6, and a 10% attrition allowance. However, these parameters could not be retrospectively specified and provide a single endpoint-specific treatment difference and corresponding SD sufficient to reproduce the original sample-size calculation.

Accordingly, the target sample size is reported as a prospective planning decision and should not be interpreted as ensuring a specified statistical power for every assessed outcome.

### 2.3. Ethics and Regulatory Aspects

The study protocol (version dated 22 February 2022) was prospectively approved by the nationally accredited Comité de Ética de San Isidro (CESI), Instituto Médico de Alta Complejidad San Isidro, San Isidro, Argentina (Approval No. 250322-003; 25 March 2022), before participant recruitment began. The trial reached primary completion on 3 June 2024 and study completion in August 2024. The trial was retrospectively registered at ClinicalTrials.gov (NCT06840964) in 18 February 2025. All participants provided written informed consent before enrollment. The study was conducted in accordance with the Declaration of Helsinki and applicable Good Clinical Practice requirements.

### 2.4. Allocation and Blinding

Participants were randomly assigned in a 1:1 ratio to receive omega-3 or placebo. The allocation sequence was computer-generated using the Oxford Minimization and Randomization software. Treatment allocation was implemented using coded treatment packs corresponding to the randomization sequence. Participants and study personnel involved in clinical assessments remained blinded to treatment allocation throughout the intervention period.

### 2.5. Intervention

Participants assigned to the intervention group received 2 g/day of pharmaceutical-grade omega-3 ethyl esters, administered as two 1000 mg capsules containing 90% purified ethyl esters (46% EPA and 38% DHA; EPA:DHA ratio ≈1.2:1). Omega-3 supplementation was administered in addition to usual antihypertensive and lipid-lowering therapy. Participants assigned to the placebo group received two visually identical capsules containing Miglyol 812 (medium-chain triglycerides). The placebo capsules were manufactured and supplied free of charge by Gador S.A., an ANMAT-authorized pharmaceutical laboratory in Argentina. Capsules were dispensed at baseline and at weeks 4 and 8.

### 2.6. Study Procedures and Prespecified Objectives

Study assessments were conducted at baseline and at the 12-week follow-up visit. The prospectively approved protocol specified three primary objective domains: (i) cardiometabolic biochemical markers, including cholesterol profile, triglycerides, uric acid, and glucose; (ii) inflammatory biomarkers, including ferritin, high-sensitivity C-reactive protein (hs-CRP), and a leukocyte-derived inflammatory ratio; and (iii) vascular wall elasticity, assessed through pulse wave velocity (PWV). Treatment tolerability was specified as a secondary objective.

Office and 24 h ambulatory blood pressure were prospectively scheduled assessments, and blood-pressure parameters, including central systolic blood pressure, formed the basis of the original sample-size rationale. The protocol did not designate a single confirmatory primary endpoint, establish a hierarchy among individual outcomes, or specify a multiplicity-adjustment procedure. These documentary differences are presented in Supplementary Table S1.

### 2.7. Dietary Assessment

Baseline dietary habits were assessed using a self-administered questionnaire incorporating food-consumption items from the MIND dietary framework and dietary questions from the WHO STEPS instrument. The questionnaire characterized habitual dietary patterns, including the reported frequency of consumption of foods providing EPA and DHA, rather than quantifying individual EPA or DHA intake in grams per day. At the final visit, participants were asked whether their dietary habits had changed during the intervention. The baseline questionnaire was not repeated. Follow-up dietary information therefore represented a qualitative self-report of perceived changes rather than a repeated quantitative assessment of intake (Supplementary Table S2).

### 2.8. Inflammatory and Biochemical Measurements

Fasting blood samples were obtained at baseline and at the end of the 12-week intervention. Laboratory assessments included high-sensitivity C-reactive protein (hs-CRP), ferritin, complete blood count, lipid profile, and fasting glucose. The neutrophil-to-lymphocyte ratio (NLR) was calculated as the ratio of absolute neutrophil to absolute lymphocyte counts.

### 2.9. Office and Central Blood Pressure Measurements

Office brachial blood pressure was assessed under standardized resting conditions using the Mobil-O-Graph^®^ NG system (IEM GmbH, Aachen, Germany). Three consecutive measurements were obtained, and the average of the last two readings was used for analysis. Central aortic blood pressure was assessed immediately afterward under the same standardized resting conditions using the same device.

### 2.10. Ambulatory Blood Pressure Monitoring

Twenty-four-hour ambulatory blood pressure monitoring (ABPM) was performed using the Mobil-O-Graph^®^ NG system (IEM GmbH, Germany). Measurements were obtained every 15 min during daytime and every 30 min during nighttime. Individual sleep and wake periods were determined from participant diaries and were used to define daytime and nighttime periods for analysis.

### 2.11. Estimated Pulse Wave Velocity

Estimated pulse wave velocity (ePWV) was obtained using the Mobil-O-Graph^®^ NG system. The device derives PWV from oscillometric pulse-wave analysis using a proprietary algorithm that incorporates demographic and hemodynamic information. Although vascular wall elasticity and PWV were prospectively specified in the original protocol, the available measure was therefore an algorithm-derived, cuff-based estimate of vascular function rather than directly measured carotid–femoral PWV.

### 2.12. Safety

Tolerability was assessed throughout the 12-week intervention based on treatment-related adverse effects, dose modification, and treatment discontinuation. Safety was assessed during follow-up visits through clinical evaluation and laboratory monitoring.

Harms were defined as any unfavorable or unintended sign, symptom, change in health status, or medical event occurring after initiation of the study intervention, regardless of its presumed relationship to the intervention. Harms were systematically assessed at each scheduled follow-up visit by asking participants about any new symptoms, health changes, or medical events since the previous visit. Participants were also specifically questioned about potential adverse effects associated with omega-3 supplementation. In addition, any spontaneously reported event was recorded and evaluated.

### 2.13. Statistical Analysis and Data Availability

Continuous variables are presented as mean ± standard deviation (SD), and categorical variables as frequencies and percentages. Longitudinal treatment effects were evaluated using linear mixed-effects models including fixed effects for treatment group (omega-3 vs. placebo), time (baseline vs. week 12), and the group-by-time interaction, with a participant-specific random intercept to account for repeated measurements within individuals. Models were fitted using maximum likelihood estimation. The group-by-time interaction represented the principal treatment-effect test. In this two-group, two-time-point design, the interaction is equivalent to the between-group difference in change from baseline to week 12, [(week 12 − baseline)_omega-3_ − (week 12 − baseline)_placebo_], and was reported with its corresponding 95% confidence interval and *p*-value. Estimated marginal means were used to characterize model-estimated values at baseline and week 12 and for complementary within-group and between-group contrasts. Degrees of freedom for estimated marginal means and contrasts were calculated using the Kenward–Roger method.

Model assumptions were assessed by inspection of model residuals, with residual normality evaluated using the Shapiro–Wilk test and homogeneity of variance assessed using Levene’s test. Model convergence and singularity were also evaluated. Because deviations from model assumptions were observed for inflammatory biomarkers, sensitivity analyses were performed for NLR, ferritin, and hs-CRP using participants with complete baseline and week-12 measurements. These analyses included two-sided permutation tests (10,000 permutations) comparing mean individual changes (week 12 − baseline) between treatment groups and Wilcoxon rank-sum tests comparing the distributions of individual changes between groups.

Analyses were performed using all available observations for each outcome, without imputation of missing values; consequently, the number of participants and observations contributing to individual models varied according to outcome availability. Two baseline nocturnal blood pressure records were considered unreliable because systolic and diastolic values were implausibly close and were therefore treated as missing for the nocturnal systolic and diastolic blood pressure analyses. No other measurements from these participants were excluded on this basis.

In a post hoc analysis undertaken during peer review, the proportions of randomized participants with available central systolic blood-pressure and ePWV measurements were compared between treatment groups at baseline and week 12 using two-sided Fisher’s exact tests. These comparisons assessed between-group differences in measurement availability and were not considered sufficient to establish a missing-data mechanism or exclude selection effects.

As a post hoc sensitivity analysis, the primary linear mixed-effects models were repeated among participants with available baseline physical-activity data, with additional adjustment for baseline physical activity. Baseline physical activity and its interaction with time were included as fixed effects to allow longitudinal change to differ according to baseline activity status. Given the multiple prospectively defined outcomes, no formal adjustment for multiple comparisons was applied. Accordingly, results were interpreted considering the magnitude and precision of effect estimates together with nominal *p*-values. All statistical tests were two-sided, and *p* < 0.05 was considered statistically significant. Analyses were performed using R version 4.3.0 (R Foundation for Statistical Computing, Vienna, Austria), with the *lme4*, *lmerTest*, and *emmeans* packages.

Individual de-identified participant data, including the data dictionary, statistical code, and other relevant study materials, will be available from the corresponding author upon reasonable request, subject to applicable ethical and data-protection requirements.

## 3. Results

### 3.1. Participant Flow and Baseline Characteristics

**Figure 1:**
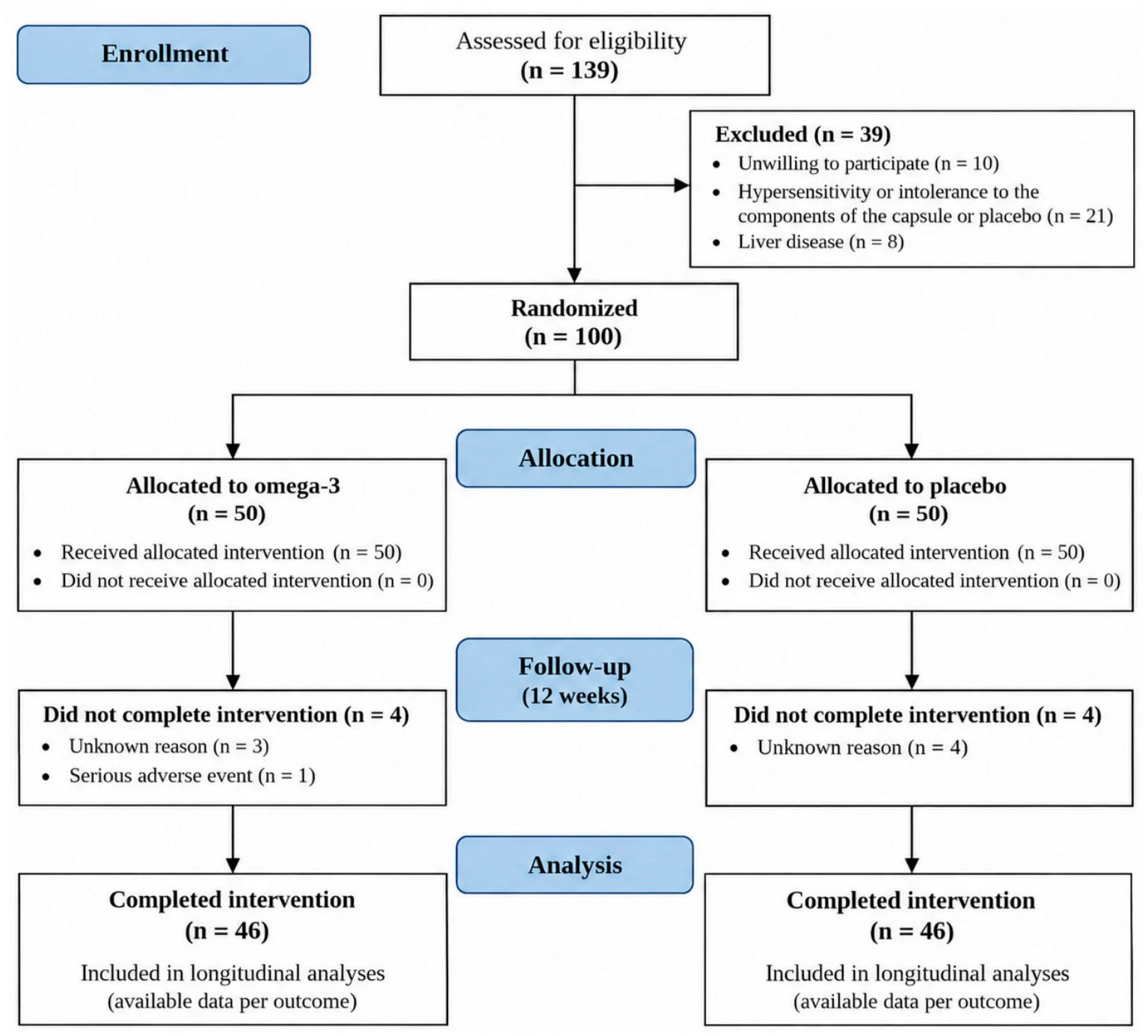
CONSORT flow diagram of participant enrollment, allocation, follow-up, and analysis. A total of 139 individuals were assessed for eligibility, of whom 100 were allocated to the omega-3 PUFA intervention group (*n* = 50) or placebo group (*n* = 50). Longitudinal analyses were conducted using available observations for each outcome.

A total of 139 individuals were assessed for eligibility. Of these, 39 were excluded before randomization: 10 declined participation, 21 had hypersensitivity or intolerance to components of the study capsules or placebo, and 8 had liver disease. The remaining 100 participants were randomized in a 1:1 ratio to receive omega-3 fatty acids (*n* = 50) or placebo (*n* = 50), and all received their allocated intervention. During the 12-week intervention, four participants in each group did not complete the intervention. In the omega-3 group, three participants discontinued for unknown reasons and one because of a severe adverse event; in the placebo group, four participants discontinued for unknown reasons. Thus, 46 participants in each group completed the 12-week intervention. Participants with available measurements contributed to the longitudinal analyses irrespective of intervention completion status, with the number of observations therefore varying across outcomes (Figure 1).

Baseline demographic and clinical characteristics are summarized in Table 1. Age, sex, anthropometric characteristics, smoking status, diabetes, antihypertensive treatment burden, and lipid-lowering treatment were generally comparable between groups. Among participants with available physical-activity data, the proportion classified as physically active was higher in the placebo group than in the omega-3 group (73.8% vs. 48.0%). Physical-activity data were unavailable for eight participants in the placebo group and none in the omega-3 group.

Baseline self-reported weekly food-consumption frequencies among participants who completed the 12-week intervention (*n* = 46 per group) are presented in Supplementary Table S2. No statistically significant between-group differences were detected in the assessed food categories. These descriptive comparisons are restricted to intervention completers and do not establish dietary comparability across the full randomized cohort or dietary stability during follow-up.

### 3.2. Inflammatory Biomarkers

**Figure 2:**
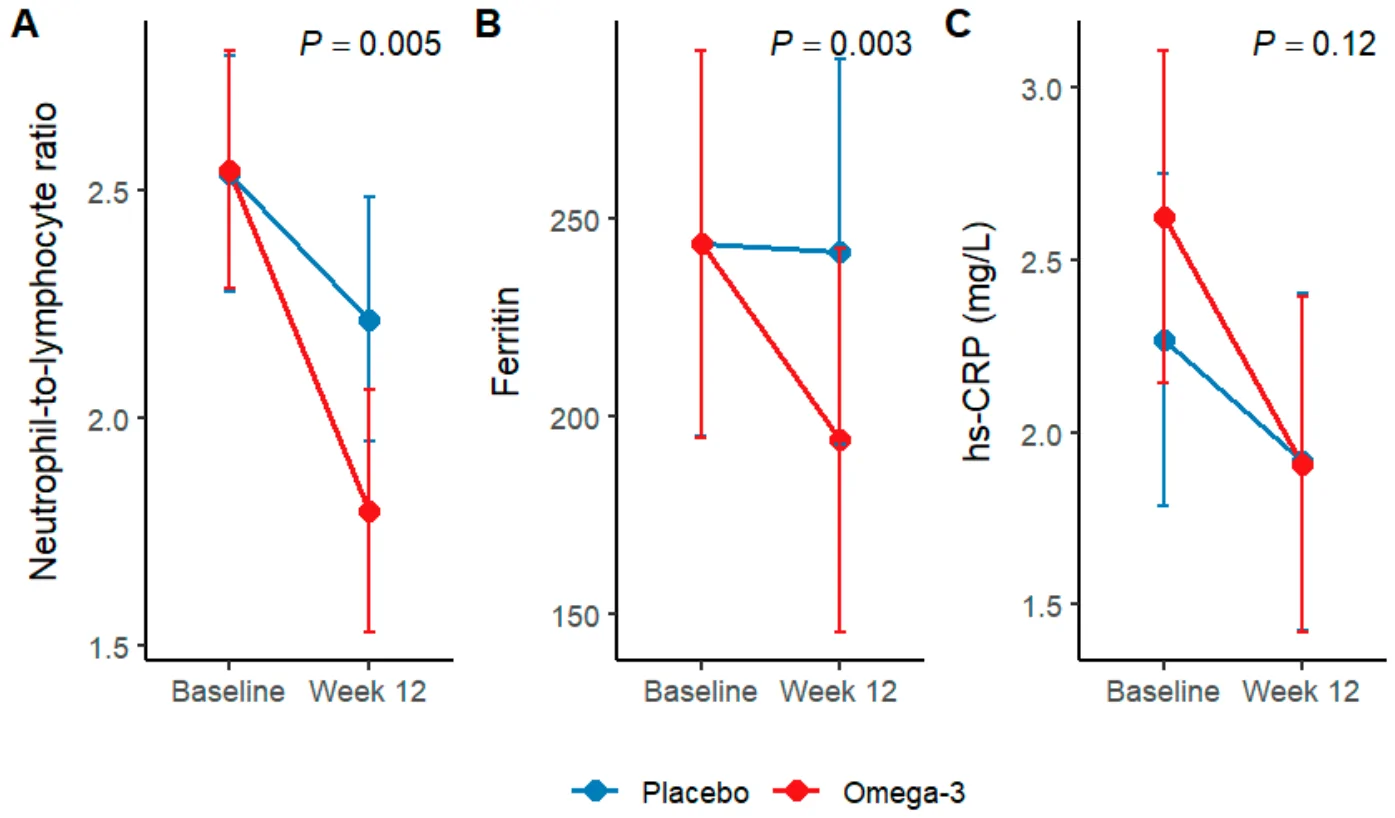
Effects of omega-3 supplementation on inflammatory biomarkers over 12 weeks. Estimated marginal means and 95% confidence intervals for (**A**) neutrophil-to-lymphocyte ratio (NLR), (**B**) ferritin, and (**C**) high-sensitivity C-reactive protein (hs-CRP) at baseline and week 12 in the placebo and omega-3 groups. Estimates were derived from linear mixed-effects models including group, time, and the group-by-time interaction, with a random intercept for participant. *p*-values correspond to the group-by-time interaction, representing the between-group difference in change from baseline to week 12. *p*-values are nominal, and 95% confidence intervals are not adjusted for multiplicity. The analyses are exploratory.

Significant group-by-time interactions were observed for NLR and ferritin, whereas the interaction was not significant for hs-CRP (Table 2 and Figure 2). The estimated between-group difference in change from baseline to week 12 was −0.43 for NLR (95% CI, −0.73 to −0.13; *p* = 0.005) and −47.32 ng/mL for ferritin (95% CI, −77.55 to −17.08; *p* = 0.003), indicating greater reductions over time in the omega-3 group than in the placebo group. In contrast, the between-group difference in change in hs-CRP was −0.37 mg/L (95% CI, −0.83 to 0.10; *p* = 0.120).

Sensitivity analyses restricted to participants with complete baseline and week-12 measurements supported the main findings. Between-group differences in individual change remained significant for NLR (permutation *p* = 0.010; Wilcoxon *p* = 0.012) and ferritin (permutation *p* = 0.003; Wilcoxon *p* = 0.019), whereas the permutation analysis remained non-significant for hs-CRP (*p* = 0.116).

In a post hoc sensitivity analysis restricted to participants with available baseline physical-activity data (*n* = 92; 42 placebo and 50 omega-3), additional adjustment for baseline physical activity and its interaction with time produced treatment-effect estimates broadly consistent with the primary analyses. Adjusted between-group differences in change were −0.40 for NLR (95% CI, −0.74 to −0.06; *p* = 0.021), −41.65 ng/mL for ferritin (95% CI, −76.82 to −6.48; *p* = 0.021), −6.99 mmHg for daytime systolic blood pressure (95% CI, −11.59 to −2.39; *p* = 0.003), and −5.39 mmHg for nighttime diastolic blood pressure (95% CI, −9.92 to −0.87; *p* = 0.020).

### 3.3. Hemodynamic Outcomes

**Figure 3:**
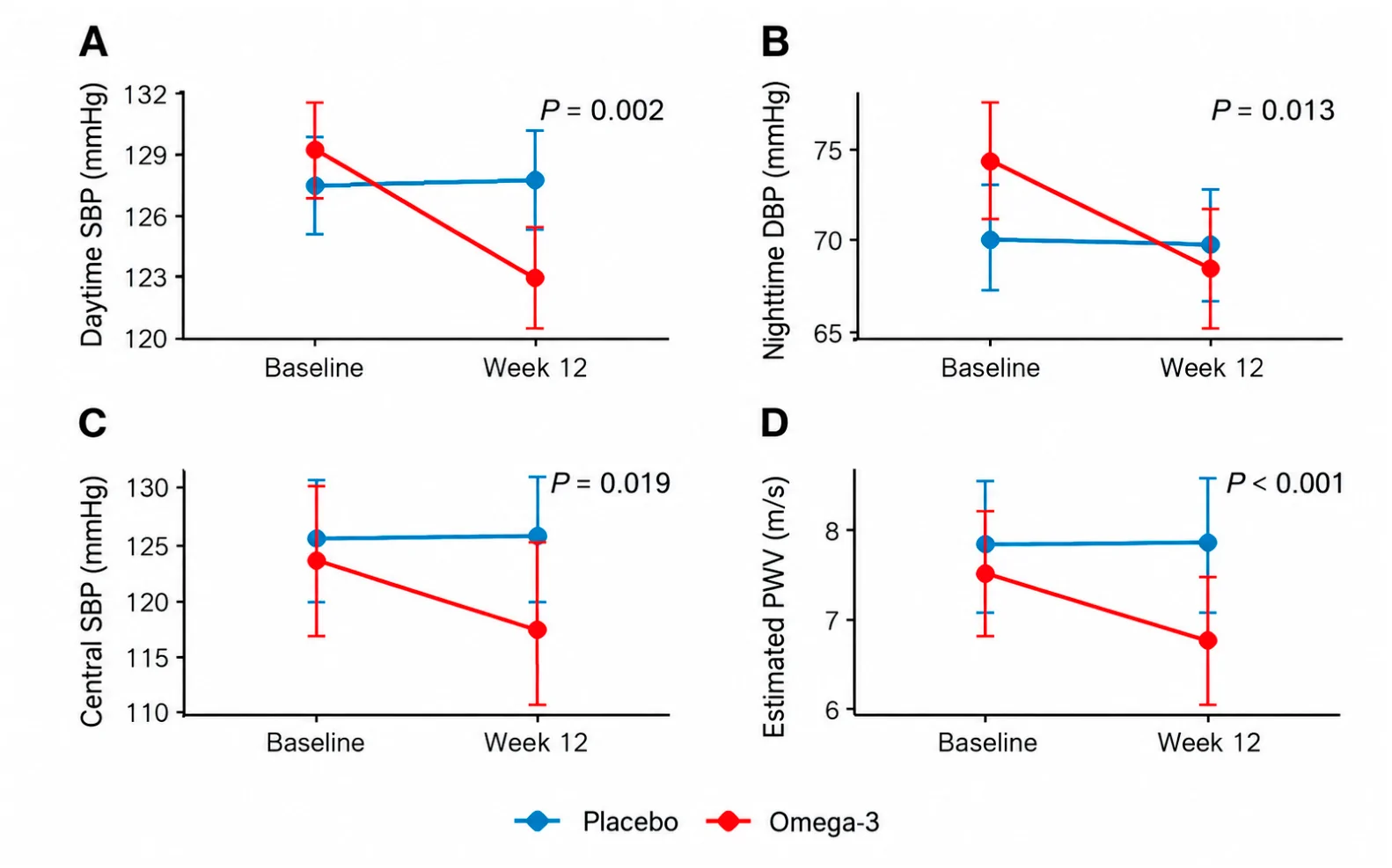
Hemodynamic and vascular outcomes showing significant group-by-time interactions over 12 weeks. Estimated marginal means and 95% confidence intervals for (**A**) daytime systolic blood pressure (SBP), (**B**) nighttime diastolic blood pressure (DBP), (**C**) central systolic blood pressure, and (**D**) estimated pulse wave velocity (ePWV) at baseline and week 12 in the placebo and omega-3 groups. Estimates were derived from linear mixed-effects models including group, time, and the group-by-time interaction, with a random intercept for participant. *p*-values correspond to the group-by-time interaction. *p*-values are nominal, and 95% confidence intervals are not adjusted for multiplicity. The analyses are exploratory.

Significant group-by-time interactions were observed for daytime systolic blood pressure, nighttime diastolic blood pressure, and central systolic blood pressure (Table 2 and Figure 3). Compared with placebo, the omega-3 group showed greater reductions from baseline to week 12 in daytime systolic blood pressure (between-group difference in change, −6.52 mmHg; 95% CI, −10.66 to −2.39; *p* = 0.002), nighttime diastolic blood pressure (−5.55 mmHg; 95% CI, −9.87 to −1.22; *p* = 0.013), and central systolic blood pressure (−6.71 mmHg; 95% CI, −12.22 to −1.20; *p* = 0.019).

A complete set of hemodynamic outcomes is presented in Figure S1 in Supplementary Materials.

Central systolic blood pressure measurements were available for 11 omega-3 and 17 placebo participants at baseline and for 8 and 12 participants, respectively, at week 12. There was no evidence of a between-group difference in measurement availability at baseline (Fisher’s exact *p* = 0.265) or week 12 (*p* = 0.454).

No significant group-by-time interactions were observed for office brachial systolic or diastolic blood pressure, daytime diastolic blood pressure, or nighttime systolic blood pressure (Table 2).

### 3.4. Vascular Outcome

A significant group-by-time interaction was observed for ePWV (Table 2 and Figure 3). The estimated between-group difference in change from baseline to week 12 was −0.77 m/s (95% CI, −1.19 to −0.36; *p* < 0.001), indicating a greater reduction in the omega-3 group compared with placebo. The corresponding treatment-effect estimate is also shown in Supplementary Figure S1. ePWV measurements were available for 27 omega-3 and 24 placebo participants at baseline and for 23 and 18 participants, respectively, at week 12. One participant had a week-12 measurement without a corresponding baseline measurement. There was no evidence of a between-group difference in measurement availability at baseline (Fisher’s exact *p* = 0.689) or week 12 (*p* = 0.416).

### 3.5. Cardiometabolic Outcomes

No significant group-by-time interactions were observed for fasting glucose, triglycerides, LDL cholesterol, or HDL cholesterol (Table 2; all *p* > 0.05).

### 3.6. Tolerability and Safety

One participant in the omega-3 group discontinued the study intervention following hospitalization for cholecystectomy, which had not been planned before enrollment. The event met the seriousness criterion of hospitalization and was not considered related to the study intervention. The participant underwent surgery and was clinically stable without complications at follow-up. No participant in the placebo group discontinued the intervention because of an adverse event.

Three additional participants in the omega-3 group and four in the placebo group discontinued for unknown reasons (Figure 1).

## 4. Discussion

In this exploratory randomized, double-blind, placebo-controlled trial, 12 weeks of omega-3 supplementation at 2 g/day added to usual pharmacological treatment in patients with hypertension and dyslipidemia was associated with differential changes in selected inflammatory, hemodynamic, and vascular outcomes compared with placebo. Significant group-by-time interactions were observed for NLR and ferritin. Omega-3 supplementation was also associated with greater reductions in daytime systolic blood pressure, nighttime diastolic blood pressure, central systolic blood pressure, and ePWV.

The reduction in NLR is consistent with emerging evidence linking omega-3 status and supplementation with the balance between circulating innate and adaptive immune cells. NLR integrates neutrophil and lymphocyte counts and has been increasingly investigated as a marker of systemic inflammatory status. The Omega-3 Index, defined as the sum of eicosapentaenoic acid (EPA) and docosahexaenoic acid (DHA) in erythrocyte membranes expressed as a percentage of total erythrocyte fatty acids, is an objective biomarker of long-term omega-3 status. Although the Omega-3 Index was not measured in the present study, evidence based on this biomarker provides relevant external context for interpreting the observed change in NLR. In a large observational study including 28,871 adults, the Omega-3 Index was inversely associated with NLR even after adjustment for age, sex, body mass index, and CRP [9]. A recent individual-level analysis pooling three randomized placebo-controlled trials and one omega-3 supplementation trial, comprising 436 participants, showed that EPA + DHA supplementation increased the Omega-3 Index and reduced NLR relative to control, with changes in omega-3 status inversely associated with changes in NLR [10]. The between-group difference observed in the present study (−0.43; 95% CI, −0.73 to −0.13) is therefore consistent with previous evidence and extends these observations to pharmacologically treated patients with coexisting hypertension and dyslipidemia.

Ferritin showed a similarly clear differential response, with a between-group difference in change of −47.32 ng/mL (95% CI, −77.55 to −17.08). Evidence specifically examining ferritin responses to omega-3 supplementation is considerably more limited than that available for CRP and inflammatory cytokines. Moreover, ferritin is not a specific inflammatory marker but an iron-storage protein that also behaves as an acute-phase reactant and can be influenced by metabolic, hepatic, inflammatory, and iron-related processes. Its reduction should therefore not be interpreted in isolation as evidence of reduced systemic inflammation. In the present study, however, the concurrent differential reduction in NLR and the consistency of both NLR and ferritin findings in permutation and rank-based sensitivity analyses support the presence of changes in selected inflammation-related biomarkers rather than reliance on ferritin alone.

In contrast, hs-CRP did not show a significant differential response. This distinction is important because it argues against interpreting the present findings as evidence of a generalized anti-inflammatory effect. An umbrella meta-analysis of 32 meta-analyses found that n-3 PUFA supplementation reduced CRP, TNF-α, and IL-6 on average, but also reported substantial heterogeneity for these outcomes [6]. Similarly, a network meta-analysis of randomized trials found no clear differential effect of EPA versus DHA on CRP, IL-6, or TNF-α and documented considerable variability across trials [11]. This heterogeneity is compatible with the multiple pathways through which EPA and DHA may influence inflammatory processes, including membrane composition, inflammatory signaling and the generation of specialized pro-resolving mediators [3,4].

The hemodynamic findings are broadly consistent with previous evidence supporting a modest blood-pressure-lowering effect of long-chain omega-3 fatty acids. A dose–response meta-analysis of 71 randomized controlled trials involving 4973 participants found a nonlinear relationship between EPA + DHA intake and blood pressure, with approximately 2–3 g/day associated with the greatest average reductions and evidence suggesting greater responsiveness among hypertensive and hyperlipidemic populations [5]. The 2 g/day dose used in our study falls within this range. The −6.52 mmHg differential change in daytime systolic blood pressure observed in our trial was larger than the average effect reported across the heterogeneous populations included in that meta-analysis. This may partly reflect the clinical characteristics of our population, although the magnitude and confidence interval of the present estimate should be considered when comparing it with pooled treatment effects.

The ambulatory findings are particularly relevant because hemodynamic effects may not necessarily be captured by conventional office measurements. In an earlier randomized trial, DHA supplementation reduced 24 h and daytime ambulatory blood pressure in mildly hyperlipidemic men [12]. Our results showed significant differential changes that were observed in daytime systolic and nighttime diastolic blood pressure, whereas office systolic and diastolic blood pressure did not show significant group-by-time interactions. This pattern illustrates the additional information provided by 24 h monitoring and is potentially relevant given that 24 h and nighttime blood pressure are particularly strong predictors of adverse cardiovascular outcomes [7]. However, a nominally significant result for an ambulatory measure and a non-significant result for an office measure do not establish that the treatment effects differ between measurement modalities. The observed pattern is descriptive and requires confirmation in studies designed to evaluate such differences.

An additional consideration when interpreting the hemodynamic findings is the baseline imbalance in physical activity. Among participants with available data, a greater proportion of the placebo group was physically active than of the omega-3 group (73.8% vs. 48.0%). This chance imbalance is potentially relevant because exercise training has well-established blood-pressure-lowering effects. A systematic review and meta-analysis of randomized trials demonstrated significant reductions in systolic and diastolic blood pressure following exercise training [13], while a more recent network meta-analysis of 270 randomized trials involving 15,827 participants confirmed significant blood-pressure reductions across several exercise modalities [14]. Importantly for the present study, a meta-analysis of 15 randomized trials involving 910 patients with hypertension showed that exercise reduced 24 h, daytime, and nighttime ambulatory blood pressure; these benefits were also significant among participants receiving antihypertensive medication [15]. However, in a post hoc sensitivity analysis restricted to participants with available baseline physical-activity data, additional adjustment for baseline physical activity produced treatment-effect estimates that were broadly consistent with the primary analyses, including for daytime systolic and nighttime diastolic blood pressure. These findings indicate that the estimated hemodynamic effects were not substantially altered after accounting for the baseline physical-activity imbalance. Nevertheless, physical activity was not experimentally controlled, and physical-activity data were missing for eight participants, all in the placebo group. Although adjustment for baseline physical activity yielded broadly similar estimates for the reported outcomes, this analysis was restricted to participants with available activity data and cannot account for unmeasured changes in activity during follow-up. It therefore provides a sensitivity assessment of the observed chance baseline imbalance rather than proof that lifestyle-related influences were absent.

Central pressure reflects the hemodynamic load experienced by the heart and central arteries and has been associated with cardiovascular outcomes independently of peripheral systolic pressure [8]. Previous intervention evidence also provides some support for an effect of omega-3 fatty acids on central hemodynamics: Iketani et al. [16] reported a reduction in central systolic blood pressure following 1.8 g/day EPA for three months in patients with hyperlipidemia. In our study the between-group difference in change in central systolic blood pressure was −6.71 mmHg (95% CI, −12.22 to −1.20). However, these findings warrant caution because measurements were available in only 28 participants, with 20 observations at week 12. The observed difference should therefore be regarded as a preliminary signal requiring confirmation rather than definitive evidence of an effect on central hemodynamics.

A similar pattern was observed for ePWV, with a between-group difference in change of −0.77 m/s (95% CI, −1.19 to −0.36). The direction of this effect is consistent with previous evidence suggesting that omega-3 fatty acids may influence arterial stiffness. A systematic review and meta-analysis of 14 randomized controlled trials found a small but significant overall reduction in PWV with fish-oil supplementation, although no significant effect was observed for augmentation index [17]. Nevertheless, direct comparison with our results requires caution. The trials included in that meta-analysis assessed cfPWV, whereas the ePWV used in the present study was derived from oscillometric pulse-wave analysis and should not be considered equivalent to directly measured carotid–femoral PWV. Baseline ePWV measurements were available for 51 participants, and 52 participants had at least one measurement across the two visits, including one participant with a week-12 measurement but no baseline measurement. Because ePWV is algorithm-derived and incorporates demographic and hemodynamic information, the observed change should not be interpreted as independent evidence of improved arterial stiffness or as equivalent to a change in directly measured carotid–femoral PWV.

In our study, these inflammatory and hemodynamic changes occurred without significant between-group changes in triglycerides, LDL cholesterol, HDL cholesterol, or fasting glucose. Although triglyceride lowering is a well-established effect of prescription EPA + DHA, its magnitude is strongly influenced by dose and baseline triglyceride concentrations; the American Heart Association advisory particularly supports a triglyceride-lowering effect at 4 g/day, a higher dose than the 2 g/day used in our study [18]. The absence of a differential triglyceride response therefore does not necessarily conflict with the established lipid effects of omega-3 fatty acids. More importantly, the absence of differential changes in conventional lipid and glucose measures suggests that the observed inflammatory and hemodynamic findings were not accompanied by measurable changes in these cardiometabolic parameters. This pattern is compatible with the broader biological actions proposed for EPA and DHA, including effects on cell membranes, inflammatory signaling, endothelial and vascular pathways, and lipid-derived pro-resolving mediators [3,4]. However, the parallel occurrence of inflammatory and hemodynamic changes should not be interpreted as evidence that one mediated the other, because the present study was not designed to test causal pathways or mediation.

Several strengths of the study should be acknowledged. We used a prospective, randomized, double-blind, placebo-controlled design and evaluated complementary aspects of the response to omega-3 supplementation, including individual inflammatory biomarkers, office and ambulatory blood pressure, central hemodynamics, and vascular function. The longitudinal mixed-effects approach incorporated all available observations rather than restricting the analyses to participants who completed the intervention. In addition, complete-case permutation and rank-based sensitivity analyses supported the NLR and ferritin findings, whereas hs-CRP remained non-significant, strengthening the distinction between positive and neutral inflammatory findings.

### Limitations

The study has several important limitations. First, multiple prospectively defined outcomes were evaluated without formal correction for multiplicity, and the approved protocol did not specify a single confirmatory primary endpoint or formal endpoint hierarchy. Individual nominally significant findings should therefore be interpreted in the context of their magnitude and precision rather than as independently confirmatory effects. Although the recruitment target was prospectively specified, the contemporaneous endpoint-specific assumptions required to reproduce the sample-size calculation could not be fully verified. The planned sample size therefore cannot be interpreted as ensuring a specified power for each assessed outcome.

Second, outcome availability differed substantially across measurements, particularly for central systolic blood pressure and ePWV, increasing susceptibility to selection effects. The overall sample size was modest, and several confidence intervals were wide, particularly for outcomes with reduced measurement availability. Moreover, ePWV was an oscillometrically derived, algorithm-based estimate that incorporates demographic and hemodynamic information rather than a direct measurement of carotid–femoral PWV.

Third, some potentially relevant behavioral and exposure-related factors could not be fully characterized. Physical activity was more prevalent in the placebo group at baseline and was incompletely recorded in that group. Although post hoc adjustment for baseline physical activity among participants with available data produced treatment-effect estimates broadly consistent with the primary analyses, physical-activity data were unavailable for eight participants, all in the placebo group. Adherence was assessed by participant report rather than by capsule counts or an objective measure of omega-3 exposure. Dietary habits were characterized at baseline, but the questionnaire was not repeated. The absence of major reported changes at the final visit does not establish quantitative dietary stability. Moreover, the assessment did not quantify background EPA and DHA exposure, and dietary intake was not experimentally controlled.

In addition, because a single EPA + DHA dose was evaluated, the study cannot establish a dose–response relationship or determine the minimum effective dose.

Fourth, the 12-week intervention permits assessment only of short-term responses and does not establish the persistence of the observed changes after supplementation, long-term safety, or effects on cardiovascular events. Participants were also recruited from a single cardiometabolic center and were receiving stable treatment for hypertension and dyslipidemia; therefore, the findings may not generalize to untreated individuals, populations with different comorbidity profiles or medication regimens, or other clinical settings.

Finally, although the study protocol and ethics approval preceded participant enrollment, trial registration occurred retrospectively after study completion. Taken together, these considerations warrant caution in interpreting individual findings and emphasize the need for prospective replication in adequately powered trials with prospectively specified endpoints.

## 5. Conclusions

In this exploratory randomized pilot trial, 12 weeks of EPA + DHA supplementation in patients with treated hypertension and dyslipidemia was associated with greater reductions in NLR and ferritin, but not hs-CRP, compared with placebo. Differential reductions were also observed in daytime systolic blood pressure, nighttime diastolic blood pressure, central systolic blood pressure, and ePWV, whereas office blood pressure and cardiometabolic biomarkers did not differ significantly between groups over time. These findings suggest a selective response to omega-3 supplementation across inflammatory, hemodynamic, and vascular domains rather than a uniform anti-inflammatory or cardiovascular effect. Given the multiplicity of outcomes and the limited availability of central hemodynamic and ePWV measurements, confirmation in adequately powered trials with prospectively specified inflammatory and vascular endpoints is warranted.

## Figures and Tables

**Table 1:** Baseline characteristics by treatment group.

Variable	Omega-3 (*n* = 50)	Placebo (*n* = 50)	*p*-Value
Demographic characteristics			
Age, years	57.70 ± 11.51	56.76 ± 10.06	0.51
Female sex, *n* (%)	23 (46%)	22 (44%)	1.00
BMI, kg/m^2^	26.5 ± 4.3	26.2 ± 4.3	0.47
Current smoker, *n* (%)	8 (16.0)	12 (24)	0.45
Former smoker, *n* (%)	4 (8)	9 (18)	0.23
Physically active, *n* (%)	24/50 (48.0)	31/42 (73.8)	0.018
Diabetes mellitus, *n* (%)	9 (18.0)	10 (20.0)	1.00
Number of antihypertensive drugs	2.14 ± 1.09	1.92 ± 0.99	0.45
Aspirin use, *n* (%)	4 (8.0)	5 (10.0)	1.00
Antidiabetic drug use, *n* (%)	9 (18.0)	10 (20.0)	1.00
Lipid-lowering treatment, *n* (%)	45 (90.0)	46 (92.0)	1.00
Fibrate use, *n* (%)	11 (22.0)	15 (30.0)	0.49
Statin use, *n* (%)	43 (86.0)	38 (76.0)	0.30

Values are mean ± SD or *n* (%), as appropriate. Percentages are calculated among participants with available data. Physical-activity data were available for 50 participants in the omega-3 group and 42 in the placebo group. Abbreviations: BMI, body mass index; SD, standard deviation.

**Table 2:** Outcomes at baseline and week 12 by treatment group. Values are presented as mean ± SD (*n*), where *n* represents the number of available observations for each treatment group and time point. Between-group differences in change and *p*-values for the group × time interaction were estimated using linear mixed-effects models. *p*-values are nominal, and 95% confidence intervals are not adjusted for multiplicity. The analyses are exploratory.

Outcome	Placebo		Omega-3		Between-Group Difference in Change (95% CI)	Nominal *p*-Value
	Baseline	Week 12	Baseline	Week 12		
Inflammatory biomarkers						
NLR	2.53 ± 1.06 (*n* = 48)	2.28 ± 0.88 (*n* = 42)	2.54 ± 0.96 (*n* = 47)	1.82 ± 0.68 (*n* = 44)	−0.43 (−0.73 to −0.13)	0.005
Ferritin, ng/mL	239.15 ± 197.80 (*n* = 46)	251.36 ± 183.28 (*n* = 44)	241.04 ± 163.42 (*n* = 45)	194.04 ± 116.14 (*n* = 47)	−47.32 (−77.55 to −17.08)	0.003
hs-CRP, mg/L	2.27 ± 1.44 (*n* = 47)	1.91 ± 1.10 (*n* = 44)	2.63 ± 2.33 (*n* = 47)	1.94 ± 1.56 (*n* = 45)	−0.37 (−0.83 to 0.10)	0.12
Hemodynamic outcomes						
Office brachial SBP, mmHg	138.76 ± 16.98 (*n* = 49)	133.46 ± 13.66 (*n* = 48)	141.69 ± 20.27 (*n* = 49)	132.00 ± 13.61 (*n* = 48)	−4.63 (−11.33 to 2.07)	0.174
Office brachial DBP, mmHg	83.30 ± 10.21 (*n* = 49)	81.77 ± 11.52 (*n* = 48)	85.85 ± 13.71 (*n* = 49)	83.32 ± 8.87 (*n* = 47)	−1.32 (−5.69 to 3.04)	0.549
Daytime SBP, mmHg	127.41 ± 7.65 (*n* = 49)	127.51 ± 8.02 (*n* = 45)	129.14 ± 10.19 (*n* = 49)	123.14 ± 7.02 (n = 44)	−6.52 (−10.66 to −2.39)	0.002
Daytime DBP, mmHg	80.77 ± 8.72 (*n* = 48)	78.09 ± 8.09 (*n* = 43)	79.33 ± 10.00 (*n* = 45)	75.02 ± 8.51 (*n* = 42)	−1.94 (−5.84 to 1.97)	0.327
Nighttime SBP, mmHg	117.24 ± 10.21 (*n* = 47)	115.15 ± 10.52 (*n* = 41)	117.65 ± 9.59 (*n* = 46)	114.09 ± 13.54 (*n* = 44)	−1.23 (−5.79 to 3.34)	0.595
Nighttime DBP, mmHg	70.17 ± 10.47 (*n* = 48)	69.66 ± 8.24 (*n* = 41)	74.45 ± 13.27 (*n* = 38)	69.18 ± 7.69 (*n* = 36)	−5.55 (−9.87 to −1.22)	0.013
Central SBP, mmHg	125.82 ± 14.00 (*n* = 17)	120.50 ± 7.10 (*n* = 12)	123.73 ± 11.61 (*n* = 11)	118.38 ± 8.37 (*n* = 8)	−6.71 (−12.22 to −1.20)	0.019
Vascular outcome						
ePWV, m/s	7.83 ± 1.77 (*n* = 24)	7.83 ± 2.12 (*n* = 18)	7.54 ± 1.93 (*n* = 27)	6.76 ± 1.92 (*n* = 23)	−0.77 (−1.19 to −0.36)	<0.001
Cardiometabolic outcomes						
Fasting glucose, mg/dL	97.02 ± 21.88 (*n* = 49)	95.96 ± 20.48 (*n* = 46)	99.20 ± 18.36 (*n* = 50)	98.11 ± 17.96 (*n* = 47)	0.41 (−3.19 to 4.01)	0.824
Triglycerides, mg/dL	150.49 ± 50.51 (*n* = 49)	144.62 ± 42.40 (*n* = 48)	151.30 ± 63.94 (*n* = 50)	145.91 ± 52.08 (*n* = 47)	0.37 (−12.39 to 13.12)	0.954
LDL cholesterol, mg/dL	107.49 ± 25.58 (*n* = 49)	103.21 ± 21.31 (*n* = 48)	106.00 ± 30.23 (*n* = 50)	102.09 ± 24.79 (*n* = 47)	−0.67 (−7.15 to 5.80)	0.837
HDL cholesterol, mg/dL	55.65 ± 17.89 (*n* = 49)	57.00 ± 17.88 (*n* = 48)	56.10 ± 17.37 (*n* = 50)	58.55 ± 16.86 (*n* = 47)	0.55 (−1.32 to 2.43)	0.561

Values are observed as mean ± SD; *n* denotes available observations at each visit. Treatment effects are group-by-time interactions from linear mixed-effects models fitted to all available observations without imputation. Negative estimates indicate a greater reduction with omega-3 than placebo. *p*-values and 95% confidence intervals are unadjusted for multiplicity; analyses are exploratory. Abbreviations: CI, confidence interval; DBP, diastolic blood pressure; ePWV, estimated pulse wave velocity; HDL, high-density lipoprotein; hs-CRP, high-sensitivity C-reactive protein; LDL, low-density lipoprotein; NLR, neutrophil-to-lymphocyte ratio; SBP, systolic blood pressure; SD, standard deviation.

## Data Availability

De-identified participant-level data covering baseline characteristics and measured study outcomes, together with a data dictionary and the statistical code supporting the reported analyses, will be available from the corresponding author upon reasonable request, subject to applicable ethical and data-protection requirements.

## Supplementary Materials

The following supporting information can be downloaded at: https://www.mdpi.com/article/10.3390/nu18213717/s1, Figure S1: Group-by-time treatment effects for hemodynamic and vascular outcomes; Table S1: Protocol to manuscript concordance; Table S2. Baseline weekly food-consumption frequencies by treatment group: Table S2a. Weekly Frequency of Omega-3 Rich Food Intake; Table S2b. Relative Percentage Contribution (%) of Each Food Source to Total Omega-3 Intake.
