# Supplementary material for: Effects of Omega-3 Supplementation on Inflammatory Biomarkers and Ambulatory and Central Blood Pressure in Patients Treated for Hypertension and Dyslipidemia: A Randomized, Double-Blind, Placebo-Controlled Trial
Source: Nutrients. 2026 Nov 9;18(21):3717. doi: 10.3390/nu18213717 (PMC13765785; doi:10.3390/nu18213717)
Supplement: Supplementary file 1 [file nutrients-18-03717-s001.zip › FIGURE S1.Group by time treatment effects for hemodynamic and vascular outcomes.pdf]

Supplementary Figure S1. Group-by-time treatment effects for haemodynamic and vascular outcomes.

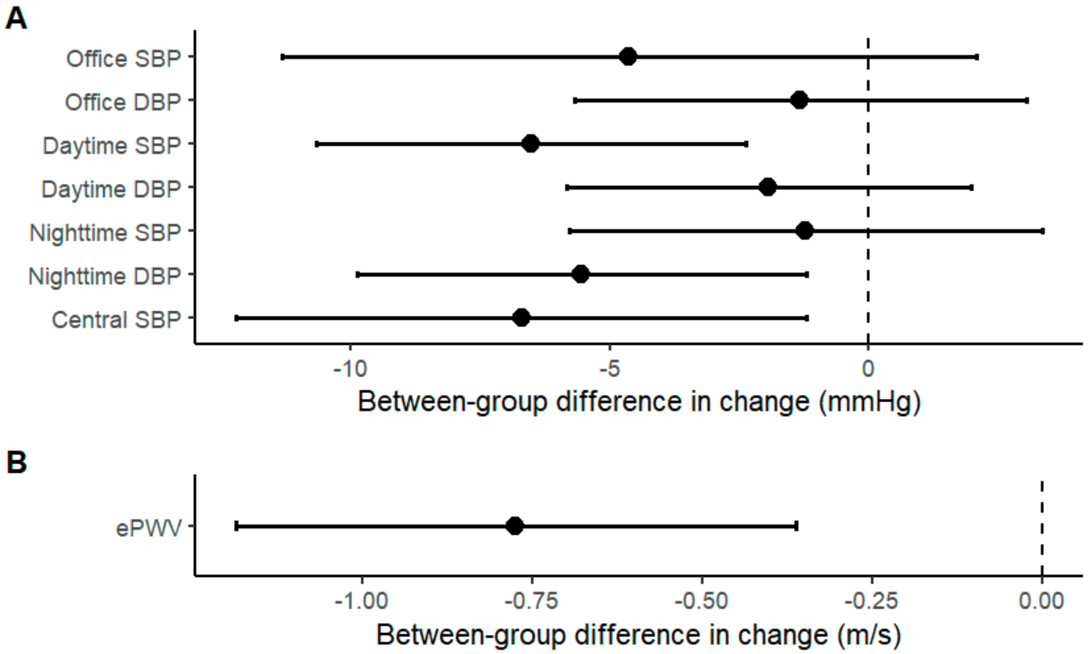

Forest plot showing the estimated between-group differences in change from baseline to week 12 and corresponding 95% confidence intervals for office, ambulatory, and central blood pressure and ePWV outcomes. Points represent the estimated between-group differences in change and horizontal bars represent their 95% confidence intervals. The vertical dashed line at zero represents no between-group difference in change; confidence intervals crossing zero indicate a non-significant group-by-time interaction at the 0.05 level. Negative values indicate a greater reduction from baseline to week 12 in the omega-3 group compared with placebo.
